# Incisional hernia after 2498 single-port access (SPA) gynecologic surgery over a 10-year period

**DOI:** 10.1038/s41598-020-74471-5

**Published:** 2020-10-15

**Authors:** Joseph J. Noh, Tae-Hyun Kim, Chul-Jung Kim, Tae-Joong Kim

**Affiliations:** 1 Division of Gynecologic Oncology, Department of Obstetrics and Gynecology, Samsung Medical Center, Sungkyunkwan University School of Medicine, 81, Irwon-ro, Gangnam-gu, Seoul, South Korea; 2 Department of Obstetrics and Gynecology, Konyang University Hospital, Konyang University School of Medicine, Taejon, South Korea

**Keywords:** Anatomy, Risk factors

## Abstract

The present study was conducted to report the perioperative outcomes of single-port access (SPA) laparoscopic gynecologic surgeries with focus on the incidence of postoperative incisional hernia from our cumulative data of 2498 patients. A retrospective review was performed on the women who had received SPA surgeries from 2008 to 2018. Patient characteristics and perioperative outcomes including the incidence of postoperative incisional hernia were analyzed. There were 2498 Korean patients who received SPA surgeries for various gynecologic diseases. The median age of the patients was 40.3 ± 9.2 years, and the mean body mass index (BMI) was 22.6 ± 3.2 kg/m^2^. A total of 3 postoperative incisional hernia occurred during the study period. Two patients whose fascial layers were closed in running sutures developed hernias 6 and 8 months after their operations. One patient whose fascial layers were closed in interrupted sutures developed hernia 11 months after her operation. The incidence of postoperative incisional hernia following SPA surgery is low in Asian women whose BMI is relatively lower than other patient populations. Interrupted suture technique may reduce postoperative incisional hernia by providing a distinct visualization of fascial layers during closure. Detailed descriptions of our surgical techniques of closing the port incision are provided.

## Introduction

The laparoscopic approach is now considered the standard surgical care for many gynecologic diseases. With the advances in surgical equipment such as articulating forceps, energy devices and barbed suture materials, single-port access (SPA) laparoscopic surgery, in which only one incision is made at the umbilicus for pelvic entry, has been adopted widely. Studies have reported the safety, better surgical outcomes and cosmetic benefits of SPA surgery^1–3^. However, despite the widespread implementation of SPA surgery, surgeons have consistently expressed concerns of postoperative incisional hernia due to relatively larger incision compared to conventional trocar insertion sites.

Previous studies have reported varying rates of umbilical hernia after SPA surgery. Early studies had established umbilical hernia rates as low as 1.5% for laparoscopic surgery when using the Hasson technique^4^. A more recent prospective study, however, reported a rate of 25.9% when following patients regularly for 3 years with physical exam and ultrasound^5^. Among other surgical specialties performing SPA surgeries, incisional hernia rates as high as 33% at 3.5 years have been reported, with increasing age and body mass index (BMI) as significant predictors for hernia development^6–9^. Theoretical mechanisms of hernia formation are the weak structure of the umbilical ring and the midline of the abdominal wall, and the larger incision of the abdominal fascia compared to conventional laparoscopic incisions^5,10^.

We previously reported our experience on surgical complications in 515 SPA surgeries^11^. Since then, we have continuously performed SPA surgeries and further data on surgical outcomes as well as surgery-related complications were collected. In the present study, we analyzed the perioperative outcomes with focus on the incidence of postoperative incisional hernia from our cumulative data of 2498 SPA surgeries. We also provide detailed descriptions of our closure methods.

## Materials and methods

### Patients

All SPA laparoscopic surgeries performed between May 2008 and December 2018 were reviewed. The surgeries were performed by a single surgeon (TJ Kim) from the beginning of the study period until February 2015. From March 2015 until the end of the study period, the operations were performed by two surgeons—TJ Kim and TH Kim who had finished her surgical training as a gynecologic oncologist under TJ Kim. The data include 2261 patients who underwent SPA surgeries by TJ Kim and 237 patients who underwent SPA surgeries by TH Kim. The surgical methods were identical between the two surgeons. The study time period includes the initial experience of SPA surgeries and the development period of a consistent surgical techniques. Patient data recorded include age, BMI, number of previous abdominal surgeries, types of previous abdominal surgeries including incision methods, total operation time and estimated blood loss (EBL). Conversions from SPA to multiport or laparotomy were also documented. The inclusion criteria were all patients undergoing SPA surgeries during the study period. The exclusion criteria were as follows: patients with previous diagnosis of ventral hernia and those with history of mesh placement in the umbilical or upper abdomen. Approvals from the Samsung Medical Center Institutional Review Board was obtained (IRB numbers: 2019-11-017).

### Study design and protocols

The decision to offer a patient a SPA surgery versus a multiport surgery was made by the attending surgeon through discussion with each patient. In general, the patients with following conditions were discouraged for SPA surgeries: patients suspected to have severe pelvic adhesions due to previous radiation or inflammatory pelvic disease, patients with large leiomyomas (usually larger than 10 cm in diameter), and patients with multiple leiomyomas who were unlikely to achieve the removal of all tumors by SPA surgery due to their locations. All patients underwent SPA surgeries under general anesthesia. Antibiotic prophylaxis with 1 g cefazolin was administered routinely 30 min before the skin incision. Repeated intraoperative doses of antibiotics were also given for prolonged operations, obese patients, and in cases with severe blood loss at anesthesiologists’ discretion. The total operative time was defined as the time from the beginning of skin incision to the completion of skin closure. EBL was calculated by subtracting the volume of irrigating fluid from the volume of total fluids collected in the suction apparatus after surgery.

Data were collected for postoperative complications, which included re-operations for any surgery-related causes, unplanned intensive care unit admission, ureteral or bladder injury, bowel injury, vaginal cuff dehiscence, deep wound infection, incisional cellulitis, blood transfusion and postoperative incisional hernia at the umbilicus. Incisional hernia was defined as any hernia that was detected clinically during postoperative surveillance either via physical examination or radiographically. Late complications were defined as any surgery-related complications that occurred one month after operations. The postoperative complications were assessed in the outpatient clinic 7 days after the surgery. Then the subsequent follow-up was scheduled according to the type of surgery performed. For those who underwent hysterectomy, the second follow-up was scheduled 30 days after the surgery whereas those who required hormonal therapy, the follow-up schedules were planned more frequently. If there were no complications during the first three follow-up visits, most women were scheduled for routine gynecologic checkup on an annual basis. The complication severity was reported using the Clavien–Dindo classification^12^. The evaluations of surgical-site infection (SSI) was based on the Centers for Disease Control and Prevention Definitions.

### Surgical methods

The SPA laparoscopic surgeries were performed in the same surgical procedures and steps by the two surgeons. After incising the skin at about 2.0–2.5 cm, subcutaneous tissue and anterior abdominal fascia were opened by Bovie electrocauterization in 40-W, monopolar coagulation mode (Bovie Medical Corporation, Inc., Melville, NY, USA) using the open Hasson technique. Entering the peritoneum, a single-port access was created by inserting a polyurethane multi-channel single-port system. The previously described platform which consisted of a wound retractor and a surgical glove was used during the earlier period of the study^13,14^, then it was replaced by a number of commercial platforms including The One Port (LapaKorea, Inc., Seoul, South Korea), OCTO Port (DalimSurgNet, Inc., Seoul, South Korea), SILS Port (Covidien, Inc., Norwalk, CT, USA) and LabSingle (Sejong Medical, Inc., Paju, South Korea). The carbon dioxide pneumoperitoneum was kept at 13 mmHg throughout the operations. The instruments used during the operations included monopolar scissors, laparoscopic energy devices such as ENSEAL (Ethicon, Inc., Somerville, NJ, USA), THUNDERBEAT (Olympus, Inc., Tokyo, Japan), or LigaSure (Medtronic, Inc., Minneapolis, MN, USA), myoma screws, laparoscopic needle holders and articulating graspers (Roticulator, Covidien, Inc., Norwalk, CT, USA).

### Closing technique

**Figure 1:**
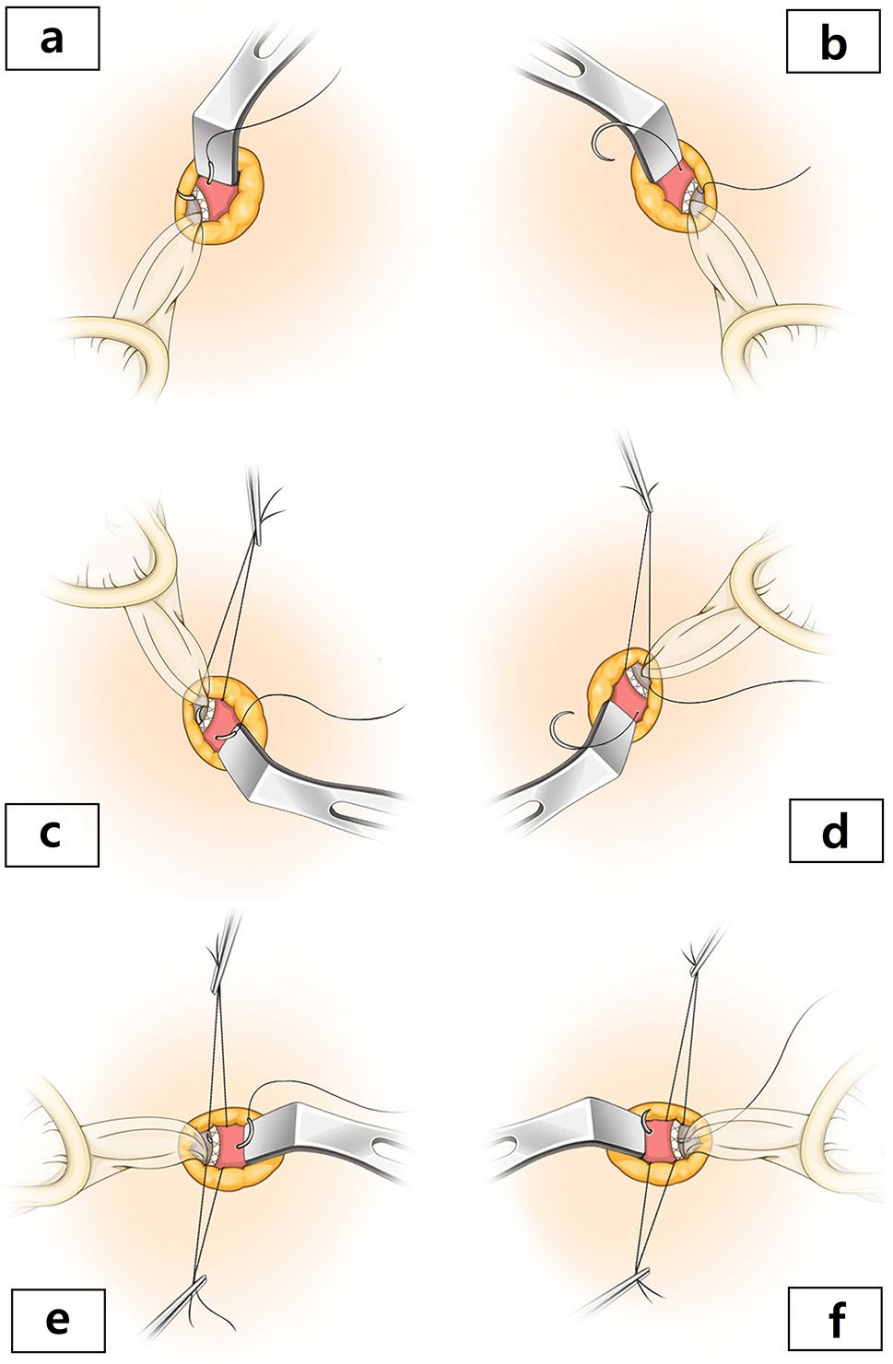
Illustration of the closing methods in single-port access laparoscopic surgery: (**a**) with gently pulling the wound retractor towards the contralateral side of the stitch, clear visualization of fascial layer is possible, and the upper end of the incision is sutured, (**b**) the other side of the fascia is sutured by pulling the wound retractor contralaterally in a similar manner, (**c**–**d**) the lower part of the incision is sutured in a similar manner, (**e**,**f**) with the sutures in the upper and lower part of the incision remained untied, the middle portion of the incision is sutured similarly.

**Figure 2:**
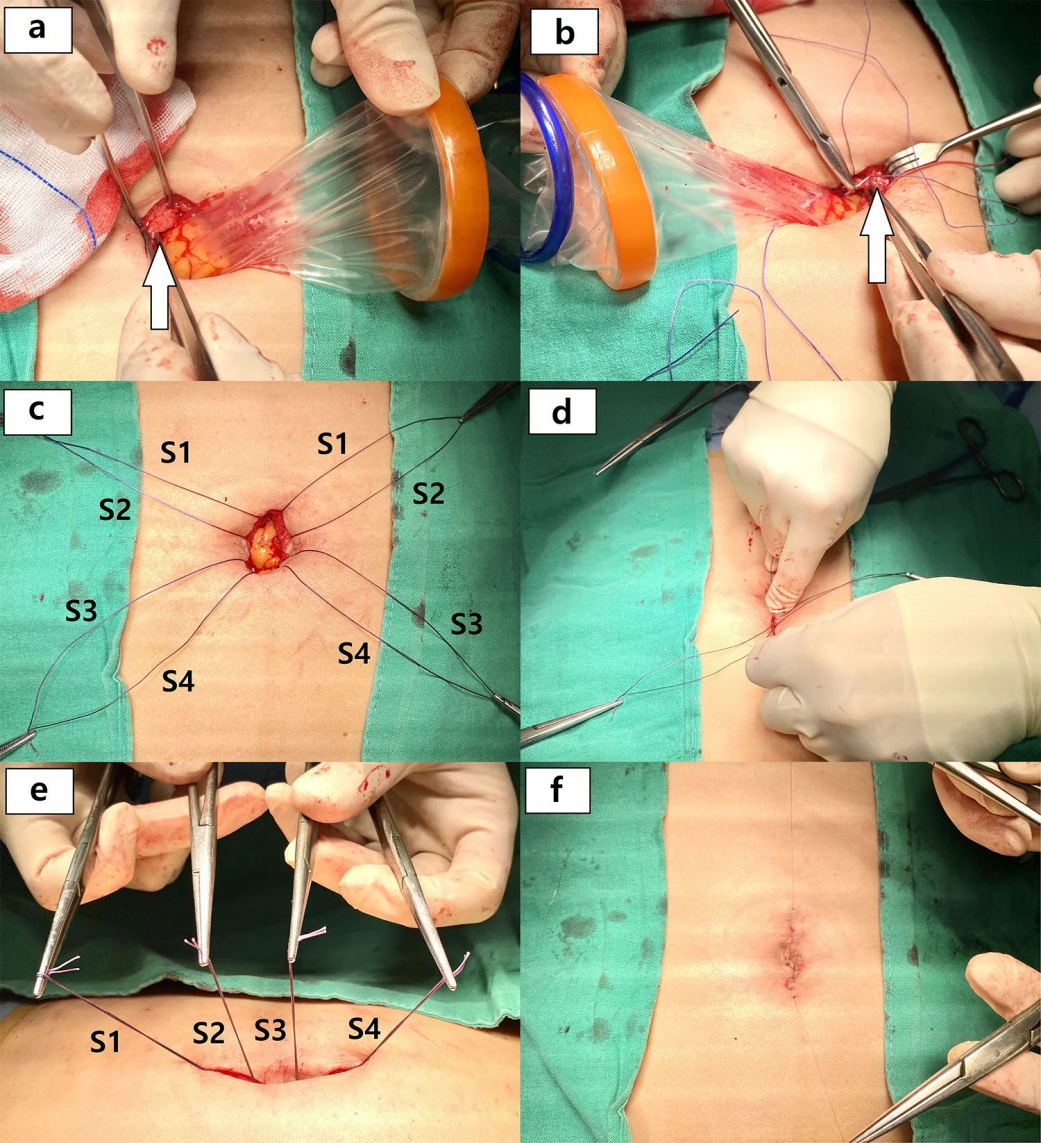
Demonstration of the closing methods: (**a**) the wound retractor is pulled towards the right side of the photo while the surgeon identifies the left upper side of the fascia (white arrow), (**b**) the wound retractor is pulled towards the left side and the surgeon throws a suture with clear visualization of the fascial layer of the opposite side (white arrow), (**c**) four interrupted sutures are completed without knots tied (S1 through S4 from cranial to caudal), (**d**) knots are tied sequentially from outer side of the incision, (**e**) tied suture materials are recognized (left: cranial, right: caudal), (**f**) after approximating subcutaneous tissue, skin is closed with absorbable materials by subcuticular suture.

Our closing technique underwent evolution during the study period. It changed from running suture of the fascial layer during the early period of the study to interrupted suture. The change occurred after the first 515 operations. Nevertheless, careful closure of the fascial incision was performed by the surgeons throughout the entire study period to prevent port-site hernia. After the completion of the laparoscopic surgeries, the inserted port was not removed but kept in situ while surgeons clearly identifying the fascial layer (Fig. 1). By gently pulling the remained port system towards one lateral side, surgeons were able to identify the fascial layer of the other lateral side more clearly, thereby throwing distinct and proper stitches (Fig. 2a,b). Sutures were done in an interrupted manner. The surgeons did not tie knots after each suture, but left them untied until all stitches were thrown through the whole length of the incision (Fig. 2c). Meticulous efforts to create dense intervals between consecutive sutures were made during the closure. The wound was closed by taking bites of fascia of 5–8 mm and placing stitches every 5 mm. After the completion of all stitches, the port-system was removed. Then the knots were tied sequentially from outer side of the incision (Fig. 2d,e). The peritoneum was not closed as a separate layer. The fascial defect was closed using 2-0 Polysorb Braided Absorbable suture GU-46 (Covidien, Mansfield, MA, USA). The soft tissues were approximated using 4-0 Monocryl (Ethicon, Inc., Somerville, NJ, USA) suture in an interrupted manner. The skin was closed with the same suture materials by subcuticular suturing (Fig. 2f). A clear adhesive bandage was applied at the end (Dermabond Mini, Ethicon, Inc., Somerville, NJ, USA).

### Follow-up

At discharge, the patients were instructed to visit the present institution for any symptoms suggestive of postoperative incisional hernia such as bulging, discomfort, redness, pain or discharge of the incision site. The follow-up schedule varied according to the disease status of each individual. The patients visited the outpatient clinic at least three times postoperatively, 7 days after the operation for initial follow-up, then between 30 and 90 days after the operation for second and third follow-up. During the outpatient clinic visit, they were asked for any symptoms suggestive of complications. Gynecologic physical examination along with the evaluation of the port insertion site were performed by the attending surgeons on every patient. Routine imaging evaluation for postoperative incisional hernia was not carried out. Transabdominal ultrasonography or abdominopelvic computed tomography (CT) was only done if postoperative incisional hernia was clinically suspected.

### Statistical analysis

The data for the present study are expressed as mean ± standard deviation for continuous variable. Statistical significance was determined using *the Fisher’s exact test* for dichotomous variables and by *the independent Student’s t test* for continuous variables. *P* values less than 0.05 were considered significant. Statistical calculations were carried out with R 3.6.2^15^.

### Ethics approval

All procedures performed were in accordance with the ethical standards of the institution and with the 1964 Helsinki declaration (and its later amendments). Approvals from the Samsung Medical Center Institutional Review Board (IRB) was obtained.

### Consent to participate

As a retrospective study, formal consent was not obtained from the patients and was not required.

## Results

Between May 2008 and December 2018, a total of 2498 patients received SPA surgeries performed by the two surgeons. Among them, 2261 patients underwent various types of SPA surgeries by one surgeon during the entire study period while 237 patients received their surgeries by another surgeon from March 2015 until the end of the study period. The median age of the patients was 40.3 years old and no differences in baseline characteristics were observed between the two surgeons’ patient groups. Additional insertions of trocars occurred in 56 patients due to inadequate exposure of surgical field and difficulties in surgical manipulation. Twelve patients started with SPA surgeries but converted to laparotomy for following reasons: severe intraperitoneal adhesion, inadequate exposure of surgical field, urinary tract injury and aorta injury. The mean BMI of the patients was 22.6 kg/m^2^. The types and numbers of surgical procedures performed by SPA are described in Table 1. The most common type of surgery via single-port incision was hysterectomy which accounted for 35% of all surgeries, followed by benign ovarian cystectomy, which accounted for 27%.

**Table 1:** Baseline characteristics of the patients who underwent single-port access (SPA) laparoscopic gynecologic surgeries.

	LAVH^a^	TLH^a^	SH^a^	Adnexectomy^a^	Cystectomy^a^	Myomectomy^a^	Others^a^	Total
**Number of cases (%)**	240 (9.6%)	628 (25.1%)	52 (2.1%)	549 (22.0%)	668 (26.7%)	324 (13.0%)	37 (1.5%)	2498
**Age (years)**	45.8 ± 5.1	46.1 ± 6.2	44.0 ± 3.5	49.9 ± 11.5	32.2 ± 7.6	38.4 ± 7.2	46.6 ± 12.2	40.3 ± 9.2
**BMI (kg/m)**^2^	23.6 ± 3.0	23.0 ± 2.8	23.0 ± 3.3	23.7 ± 3.2	21.3 ± 3.6	21.8 ± 2.3	21.8 ± 2.1	22.6 ± 3.2
**Number of patients with previous abdominal surgery**	81	176	14	110	48	23	5	457
Cesarean section (Pfannenstiel’s incision)	50	138	7	87	27	16	3	328
Cesarean section (lower midline incision)	5	4	2	5	0	0	0	16
Pelviscopy	3	14	2	8	10	4	1	42
Other abdominal surgery (transverse incision)	8	0	1	2	2	0	0	13
Other abdominal surgery (lower midline incision)	3	8	0	5	2	0	0	18
Appendectomy	5	8	1	3	7	2	1	27
Tubal ligation	7	2	1	0	0	1	0	11
Others	0	2	0	0	0	0	0	2
**Total operation time (min)**	119.1 ± 46.2	94.8 ± 37.1	138.3 ± 79.9	66.9 ± 35.6	92.4 ± 41.4	117.6 ± 56.5	89.7 ± 60.7	110.0 ± 46.9
**EBL (mL)**	369.9 ± 257.4	237.7 ± 179.0	266.7 ± 141.8	65.0 ± 65.7	96.4 ± 89.2	120.6 ± 80.9	130.8 ± 142.2	233.9 ± 217.4

*LAVH* laparoscopy-assisted vaginal hysterectomy, *TLH* total laparoscopic hysterectomy, *SH* subtotal hysterectomy, *EBL* estimated blood loss.

^a^If multiple procedures were performed during one operation, following order was applied and the most representative procedure was counted (LAVH, TLH, SH, myomectomy > adnexectomy > cystectomy > others). For example, if a patient undergoes LAVH with ovarian cystectomy, the operation is counted as LAVH, not cystectomy.

We reported 11 complications out of 515 surgeries in the previous study^11^. Since then, additional 1983 cases of SPA surgeries were performed. Fifteen complications were reported during the later period including 4 urinary tract injuries, 5 postoperative bleeding, 2 small bowel injuries, 1 umbilical wound infection, 1 sigmoidovaginal fistula and 1 vessel (aorta) injury. We reported one case of vaginal vault evisceration and two cases of umbilical hernia in the previous 515 cases^11^. During the later period of the present study, we identified one additional case of umbilical hernia who underwent subtotal hysterectomy. The herniation was found 11 months after the operation during routine gynecologic checkup. However, no more patients with late complications including umbilical hernia occurred in later study periods. Brief explanation of each complication case is described in Supplementary Table S1. Among the complications listed, the three umbilical hernia cases and one aorta injury case may be considered to be related to SPA surgical techniques directly. The aorta injury occurred in a women whose BMI was 19.3. The knife injured the vessel directly when incising the fascial layer due to the short distance between the great vessels and the anterior abdominal wall. Although the surgeons did recognize many surgical complications intraoperatively, and thereby repaired them without delay, numerous complication cases were not recognized until the patients claimed symptoms or visited the emergency department after being discharged.

## Discussion

The present study reports our cumulative data of SPA surgeries in 2498 patients with focus on postoperative incisional hernia at the umbilicus. We experienced significantly less cases of incisional hernia compared to previous studies. The study represents one of the largest cases of SPA surgeries and offers a unique opportunity to learn from our collective experience.

Extensive studies have been conducted to assess the incidence and risk factors of hernia development after SPA surgery (Table 2). Reported risk factors include wound infection, obesity, patient age, and the location, type, and size of the trocar employed^16–19^. Diabetes, long duration of surgery, and the need for fascial enlargement for specimen extraction have also been suggested^5,20^. Previous studies report a wide range of variation in the incidence rates of postoperative hernia at the umbilicus. The majority of the studies have a clear and transparent description of methodology of the study design, so that the obtained results are acceptable. However, the major limitation to accurately assess the true incidence rates and risk factors is clinical heterogeneity. Studies adopted different patient populations as well as different surgical techniques to close the port site. This inevitably produces uncontrolled bias. A meta-analysis comprising 35 randomized controlled trials (RCT) and a total of 3051 patients investigated the quality of study designs on postoperative incisional hernia after SPA surgeries. The authors concluded that only 3 out of the 35 trials had an overall low risk of bias presented in their study designs^21^. Other shortcomings of the RCTs include small number of patients, which sometimes do not reach the number that can be fixed by the learning curve. High rates of patients lost to follow-up and the brevity of follow-up period were also common. It should also be considered that most studies have investigated the incidence of postoperative hernia as a secondary outcome. All of these factors contribute to the inconsistent observations obtained from previous studies and can explain the wide range of results reported so far.

**Table 2:** Previous studies on incisional hernia after SPA laparoscopic surgeries.

Year	Author	Surgery	Type of study	Number of patients	Single-port sutures	Findings
2013	Lee	Gastrectomy Gastric banding	Retrospective comparison between single-port and multiport	163 single-port 513 multiport	Absorbable interrupted sutures	1 hernia (0.6%) in single-port group (median follow-up 7 months) *vs.* 3 hernias (0.6%) in multiport group (median follow-up 18 months)
2014	Agaba	Cholecystectomy, appendectomy, sleeve gastrectomy, gastric banding, Nissen fundoplication, colectomy, gastrojejunostomy	Retrospective cohort	205 single-port	Absorbable 3 figure-of-eight sutures	6 hernias (2.8%) developed (follow-up 36 months)
2019	Hoyuela	Cholecystectomy	Retrospective comparison between single-port and multiport	45 single-port 140 multiport	Long-term absorbable monofilament running sutures	6 hernias (13.3%) in single-port group *vs.* 5 hernias (4.7%) in multiport group (mean follow-up 58.7 months)
2016	Buckley	Cholecystectomy, appendectomy, colectomy, fundoplications, inguinal herniorrhaphy, others	Retrospective cohort	787 single-port	Absorbable 2 figure-of-eight sutures	50 hernias (6.35%) developed (mean follow-up 34 months)
2017	Moulton	Hysterectomy, adnexectomy, adnexal mass removal	Retrospective cohort	898 single-port	Absorbable and non-absorbable suture materials used at the surgeon’s discretion. Suture technique is not described	50 hernias (5.5%) developed (median follow-up 37.2 months)
2019	Casaccia	Cholecystectomy	Retrospective comparison between single-port and multiport	60 single-port 60 multiport	Suture materials and technique are not described	4 hernias (7.1%) in single-port group *vs.* 1 hernia (2%) in multiport group (median follow-up 18 months)

Most laparoscopic surgeons agree that the diameter of the port incision is the single most important factor to cause port site incisional hernia. Because, in SPA surgeries, the fascial defect is larger than that in conventional laparoscopy, SPA surgeries are inherently at risk of postoperative port site hernia development. The current prevailing consensus regarding trocar or port sites is that all fascial defects greater than 10 mm should be closed^22^. SPA surgeries employ a 2.0–2.5 cm umbilical incision for insertion of the working port. Therefore, the incisional site should be closed properly in order to prevent complications. Nevertheless, questions remain regarding the optimal closure technique in the setting of multiple patient risk factors. From our cumulative experience, we concluded that continuous suturing of the umbilical port site is the least recommended way of closing the port sites. As the suture continues from one end of the incision to the other, the space between left and right fascial layer progressively becomes smaller, making it difficult for surgeons to identify fascial layer distinctly. There is a higher chance for surgeons to improperly suture the fascia especially for obese patients in which needle manipulation is difficult due to thickened abdominal wall and deep location of the umbilicus. Therefore, it is strongly recommended to have interrupted sutures and leave the incision open until all stitches are properly made on each side of the incision, then to tie the knots of all sutures at once. Descriptions of closure for SPA surgeries reported in the literature are varied. They include interrupted sutures, two or three figure-of-eight sutures, and running sutures, with both absorbable and non-absorbable suture materials. There are no good quality comparative studies investigating different suture materials or techniques for closure of port sites yet.

Ideal diagnostic modalities for incisional hernia have not been evaluated either. Most studies show that medical imaging will increase the rate of detection of incisional hernias compared to physical examination alone^23^. In an everyday clinical setting, this is usually not feasible and oftentimes unnecessary because most asymptomatic hernias do not require treatment. Computed tomography (CT) is reliable and reproducible, whereas ultrasound is more operator-dependent. However, CT scan will induce a radiation load to the patients and ultrasound is more accessible in most health care settings. Therefore, when postoperative incisional hernia is clinically suspected, it is recommended to further assess the port site with transabdominal ultrasound. A good standardization and dynamic evaluation by ultrasound of the abdominal wall such as the dynamic abdominal sonography for hernia (DASH) technique may enhance diagnostic accuracy^24^.

As shown by the results of the present study, the authors observed unusually low rates of postoperative incisional hernia during the 10-year study period. A number of important patient characteristics might be accounted for these observations. First, the patients in the present study were leaner than the patient populations investigated in other previous studies. For example, Moulton and colleagues reported 5.5% of hernia development in 898 patients while Buckley and colleagues reported 6.4% of hernia development in 787 patients after SPA surgeries (Table 2). The mean BMI of the patients in the study by Moulton was 29.6 kg/m^2^ whereas the mean BMI of the patients in the study by Buckley was 28.0 kg/m^2^. They are significantly higher than the mean BMI of the patients in the present study, which was 22.6 ± 3.2 kg/m^2^. The three patients who developed incisional hernia in the present study, indeed, all had relatively higher BMI compared to other patients (25.7, 26.6 and 24.3 kg/m^2^). Patients with low BMI allow clear visualization of the fascial layer for surgeons to identify during fascial closure, which is the most critical surgical step during port site closure to prevent postoperative hernia. A relatively lower BMI of Asian women in comparison to that of Caucasian populations may have contributed to the lower incidence of postoperative incisional hernia. The generalizability of the results obtained from the present study, thus, may be limited due to this patient characteristic. Another reason may be related to patient comorbidities. Studies have reported that hernia rates are higher among patients with increasing medical comorbidities, given that patients with American Society of Anesthesiologists (ASA) score of III or IV were predictors for hernia development^25^. The present study included patients in relatively healthy conditions (ASA score I and II) only, which might have further reduced the incidence of postoperative incisional hernia.

The present study has its strength in its large number of patients evaluated. The present institution has a high-volume of SPA surgeries as one of the biggest centers in South Korea. The surgeons and highly-trained assistants in the present study strictly followed the standardized technique to close the incision, thereby minimizing potential variations that may be induced by the provider. A number of limitations exist as well. First, the retrospective nature of the study may have led to the selection bias of patient selection. Second, no imaging modalities were utilized to diagnose incisional hernia. The postoperative follow-up visits were individualized based on disease status, and no standardized follow-up evaluation for hernia was conducted. We perform postoperative follow-up up to 90 days in routine practice unless there are any surgical or medical complications that require further management. Then the patients are usually referred to local clinics for future checkup. This may raise concerns regarding potential loss of patients who may have been treated at other institutions for postoperative incisional hernia that we were not aware of. However, South Korea has a very unique national healthcare system which provides all citizens access to secondary or tertiary medical institutions with ease. It is also a small nation geographically in which one can travel anywhere in less than three hours by public transportation. A good patient reference system has also been established among medical institutions. Therefore it is unusual for patients not to re-visit the institution from which they received primary treatment if they have any symptoms or signs suggestive of complications. Considering the unique medical environment of the place, we believe that the low incidence of incisional hernia observed in the present study would not increase significantly even if longer postoperative follow-up had been performed. Another limitation is that the patients included in the present study were generally healthier compared to the patients investigated in other studies (reflected by ASA scores). We also did not examined the comorbidities or underlying medical conditions of the patients in detail. For example, diabetes mellitus, as previous research has found, is one of the most important predisposing risk factors for incisional hernia. The patients in the present study reported whether they had diabetes or not prior to their surgical treatments. However, the severity of the disease was not assessed. The extent to which diabetes mellitus influences wound healing and consequently the incidence rates of postoperative incisional hernia must be closely related to the severity of the disease. Because many patients with diabetes mellitus were being treated at other institutions (usually local clinics) at the time of surgery, it was difficult to evaluate the disease severity with accuracy. Therefore further studies are warranted to investigate the effects of diabetes on incisional hernia rates.

It is undeniable that SPA surgery provides a significantly higher cosmetic benefit and earlier recovery of the patients than multiport laparoscopic surgeries. Nevertheless, surgeons must acknowledge that there is a potentially increased risk of surgical-site complications such as herniation in SPA surgery and that facial closure is a vital component. With the surgical techniques described in the present study, we have achieved a very low incidence rate of postoperative incisional hernia and these methods are believed to improve the surgical outcomes of future SPA surgeries.

## Supplementary information

Supplementary Table
