# Supplementary material for: Incisional hernia after 2498 single-port access (SPA) gynecologic surgery over a 10-year period
Source: Sci Rep. 2020 Oct 15;10:17388. doi: 10.1038/s41598-020-74471-5 (PMC7562698; doi:10.1038/s41598-020-74471-5)
Supplement: Supplementary file 1 — Supplementary Table [file 41598_2020_74471_MOESM1_ESM.docx]

Supplementary table 1. Perioperative complications of SPA laparoscopic gynecologic surgeries.

| **Types of complication** | **Number of patients** | **Procedures** | **Clavien-Dindo classification** | **Comments** | |
| --- | --- | --- | --- | --- | --- |
| Urinary tract injury | 7 | LAVH^a^ | IIIB | **Distal ureter injury was found on post-operative day 5. Repair was done by laparotomy.**  Age: 46, height: 161 cm, weight: 55 kg (BMI^a^: 21.4)  Operative indication: adenomyosis  Uterus weight: 350 g, operation time: 3 hours 8 minutes   - Patient was discharged on POD^a^ #3 (uneventful until discharge). - Emergency department visit on POD^a^ #5 for abdominal pain and distension. - Right hydronephrosis with distal ureteral obstruction was seen on APCT^a^. - Ureterocystostomy with psoas hitch by laparotomy was done by urologists (uneventful afterwards) | |
|  |  | LAVH^a^ | IIIB | **Intraoperative bladder injury. Repair was done immediately during operation.**  Age: 41, height: 153 cm, weight: 73 kg (BMI^a^ 31.1)  Operative indication: leiomyoma  Uterus weight: 138 g, operation time: 3 hours 3 minutes   - Posterior serosal wall of bladder was injured during bladder dissection. - Immediate repair with absorbable suture was done. - Urologists checked both ureteral orifice and their patency. - Foley catheter was kept until POD^a^ #7. Patient was discharged on POD^a^ #7 (uneventful afterwards). | |
|  |  | TLH^a^ | IIIB | **Intraoperative bladder injury. Repair was done immediately during operation.**  Age: 46, height: 157 cm, weight: 50 kg (BMI^a^ 20.3)  Operative indication: adenomyosis  Uterus weight: 502 g, operation time: 2 hours 50 minutes   - Previous operation: cesarean section twice. - Severe adhesion between uterus and bladder was seen. Adhesiolysis was performed. - Bladder serosal layer injury (about 2 cm) was noted during adhesiolysis. - Immediate repair with absorbable suture was done. - Urologists checked both orifice by cystoscopy. - Patient was discharged on POD^a^ #4 with Foley catheter in situ (Foley catheter was removed on POD^a^ #6 in outpatient clinic. Uneventful afterwards.) | |
|  |  | LAVH^a^ | IIIB | **Intraoperative ureter injury was suspected. Double-J catheter was inserted by a urologist.**  Age: 42, height: 155 cm, weight: 49 kg (BMI^a^ 20.1)  Operative indication: leiomyoma  Uterus weight: 145 g, operation time: 2 hours 43 minutes   - Left distal ureter kinking was noted during hysterectomy. No urine leakage was seen. - Left double-J catheter was inserted by urologists. - Patient was discharged on POD^a^ #3 (uneventful afterwards) | |
|  |  | TLH^a^ | IIIB | **Intraoperative bladder injury. Repair was done immediately during operation.**  Age: 40, height: 161 cm, weight: 55 kg (BMI^a^ 21.4)  Operative indication: adenomyosis  Uterus weight: 188 g, operation time: 3 hours 43 minutes   - Right posterior serosal wall of bladder was injured (about 0.5 cm). - Primary repair with absorbable suture was done. - Double-J catheter was inserted. - Foley catheter was kept until POD^a^ #7. Patient was discharged on POD^a^ #7 (uneventful afterwards) | |
|  |  | TLH^a^ | IIIB | **Bladder injury was found on post-operative day 6. Repair was done by laparoscopy.**  Age: 45, height 163 cm, weight: 52 kg (BMI^a^ 19.6)  Operative indication: leiomyoma  Uterus weight: not measured, operation time: 2 hours 30 minutes   - Hysterectomy was performed uneventfully. - Patient claimed abdominal pain and distension on POD^a^ #6. - APCT^a^ revealed abdominal fluid collection and 1 cm defect of bladder dome. - Emergent laparoscopic repair of bladder (primary repair with absorbable suture) was performed. - Patient was discharged 7 days after the repair (uneventful afterwards)   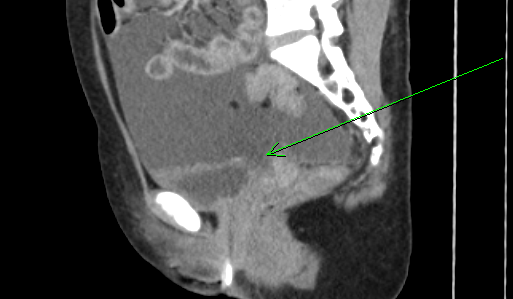  <APCT^a^ revealed 1 cm defect of bladder dome> | |
|  |  | TLH^a^ | IIIB | **Intraoperative bladder injury. Repair was done immediately during operation.**  Age: 51, height: 158 cm, weight 66 kg (BMI^a^: 26.6)  Operative indication: leiomyoma, adenomyosis  Uterus weight: 414 g, operation time: 1 hour 56 minutes   - About 1.5 cm bladder posterior serosal wall was noted during bladder detachment. - Immediate repair with absorbable suture was done. - Urologists performed cystoscopy, in which no abnormal findings were seen. - Foley catheter was kept until POD^a^ #7. Patient was discharged on POD^a^ #7 (uneventful afterwards) | |
| Vaginal vault bleeding | 1 | TLH^a^ | I | **Gauze compression was done. Hospital stay was extended.**  Age: 46, height: 163 cm, weight: 66 kg (BMI^a^ 24.9)  Operative indication: leiomyoma  Uterus weight: 358 g, operation time: 1 hour 7 minutes   - Hysterectomy was performed uneventfully. - Profuse vaginal stump bleeding was noted on POD^a^ #3. - Hemoglobin 9.8 g/dL was checked (pre-operative hemoglobin was 11.4 g/dL), vital signs remained stable. - Gauze packing was done. Hospital stay was extended until POD^a^ #7. - Bleeding stopped spontaneously. | |
| Vaginal vault abscess | 1 | LAVH^a^ | II | **Found on post-operative day 7. Single-port access laparoscopic exploration and drainage was done at another institution.**  Age: 47, height: 171 cm, weight: 105 kg (BMI^a^ 36.0)  Operative indication: adenomyosis  Uterus weight: 201 g, operation time: 4 hours 1 minute   - Hysterectomy was done uneventfully. - Fever (38.4°C) on POD^a^ #4 with vaginal discharge were noted. - WBC^a^ 9940 /µL (segmented neutrophil 79.8%), CRP^a^ 2.94 mg/dL, ESR^a^ 30 mm/hr were checked. - Single-port access laparoscopic exploration and drainage of intraabdominal fluid was done on POD #7. No significant abnormal findings were seen during the operation. - Prescribed intravenous antibiotics (ciprofloxacin) for 2 days, then oral antibiotics (ciprofloxacin) for 10 days. - Infective signs and symptoms alleviated. | |
| Vaginal vault discharge | 1 | SH^a^ | II | **Found on post-operative day 2. Transcervical drainage was done.**  Age: 46, height: 160 cm, weight: 56 kg (BMI^a^ 21.9)  Operative indication: leiomyoma  Uterus weight: 320 g, operation time: 5 hours 49 minutes   - Severe adhesion in the posterior cul-de-sac was noted. - Adhesiolysis and subtotal hysterectomy were performed. - Patient claimed abdominal pain and vaginal discharge on POD^a^ #2. Transabdominal ultrasonography revealed fluid collection in the pelvis. - Cervical dilatation and transcervical drainage of the fluid was done. Serosanguineous fluid 1300 mL was drained. Fluid creatinine was 0.52 mg/dL. No evidence of urine leakage or bleeding was seen. - Symptoms alleviated spontaneously. Patient was discharged on POD^a^ #7. | |
| Umbilical wound infection | 1 | Cystectomy | I | **Found on post-operative day 10 with wound discharge during the visit to the emergency department for sore throat and fever. Daily dressing and oral antibiotics were prescribed.**  Age: 33, height: 172 cm, weight: 65 kg (BMI^a^ 22.1)  Operative indication: mature cystic teratoma  Operation time: 2 hours 15 minutes   - Umbilical port site closure was uneventful. Patient was discharged on POD^a^ #1. - Patient visited the emergency department on POD^a^ #10 for sore throat and fever. Umbilical wound discharge (yellowish, mucoid) was noted. No dehiscence was seen. - Daily wound dressing with betadine and oral antibiotics (ceftriaxone) for 10 days were prescribed. - No discharge or any other abnormal findings were seen on POD^a^ #21. | |
| Bowel injury | 3 | LAVH^a^ | IIIB | **Intraoperative rectal injury. Repair was done immediately via vaginal approach.**  Age: 41, height: 156 cm, weight 54 kg (BMI^a^ 22.1)  Operative indication: adenomyosis  Uterus weight: 436 g, operation time: 5 hours 10 minutes   - Severe adhesion in the posterior cul-de-sac was noted. During adhesiolysis, about 1.5 cm defect of rectum (about 6 cm above the anal verge) was found. - Colorectal surgeons repaired the defect with absorbable sutures. Rectal tube was inserted for 5 days. - Patient was discharged on POD^a^ #7 (uneventful afterwards). | |
|  |  | TLH^a^ | IIIB | **Intraoperative small bowel serosal tear. Repair was done immediately by laparoscopy.**  Age: 45, height: 161 cm, weight: 64 kg (BMI^a^ 24.9)  Operative indication: adenomyosis  Uterus weight: 330 g, operation time: 1 hour 59 minutes   - Severe adhesion between small bowel and uterus anterior wall was seen. - Adhesiolysis was performed. During adhesiolysis, the serosal layer of small bowel was injured (about 1 cm). - Immediate primary repair with absorbable suture was done. - Patient was discharged on POD^a^ #3 (uneventful afterwards). | |
|  |  | TLH^a^ | IIIB | **Intraoperative small bowel serosal tear. Repair was done immediately by laparoscopy.**  Age: 58, height: 161 cm, weight: 67 kg (BMI^a^ 26.0)  Operative indication: adenomyosis  Uterus weight: 280 g, operation time: 4 hours 35 minutes   - Previous operation: cesarean section three times - Severe adhesion in the pelvic cavity was seen. Small bowel, rectum, sigmoid colon, uterus and both adnexal structures were all adhered. - Small bowel serosal wall injury was noted (about 1 cm) during adhesiolysis. - Immediate repair with absorbable suture was performed. - Patient was discharged on POD^a^ #7 (uneventful afterwards). | |
| Fistula | 2 | LAVH^a^ | IIIB | **Vesico-vaginal fistula was found on post-operative day 21. Repair was done by laparoscopy.**  Age: 50, height: 155 cm, weight: 55 kg (BMI^a^ 23.0)  Operative indication: leiomyoma  Uterus weight: 219 g, operation time: 2 hours 1 minute   - Previous operation: cesarean section twice, radical subtotal gastrectomy due to early gastric cancer - Moderate adhesion between uterus and bladder was noted. Adhesiolysis was performed. - Patient was discharged on POD #5 uneventfully. - Patient visited the emergency department for low abdominal pain and sanguineous vaginal discharge on POD^a^ #21. - APCT^a^ revealed vesico-vaginal fistula. - Vesico-vaginal fistula repair was done. Laparoscopic approach to repair bladder was done with absorbable continuous suture. Vaginal approach to repair vaginal wall defect was done with absorbable interrupted suture. - Patient was discharged 15 days after the repair uneventfully.   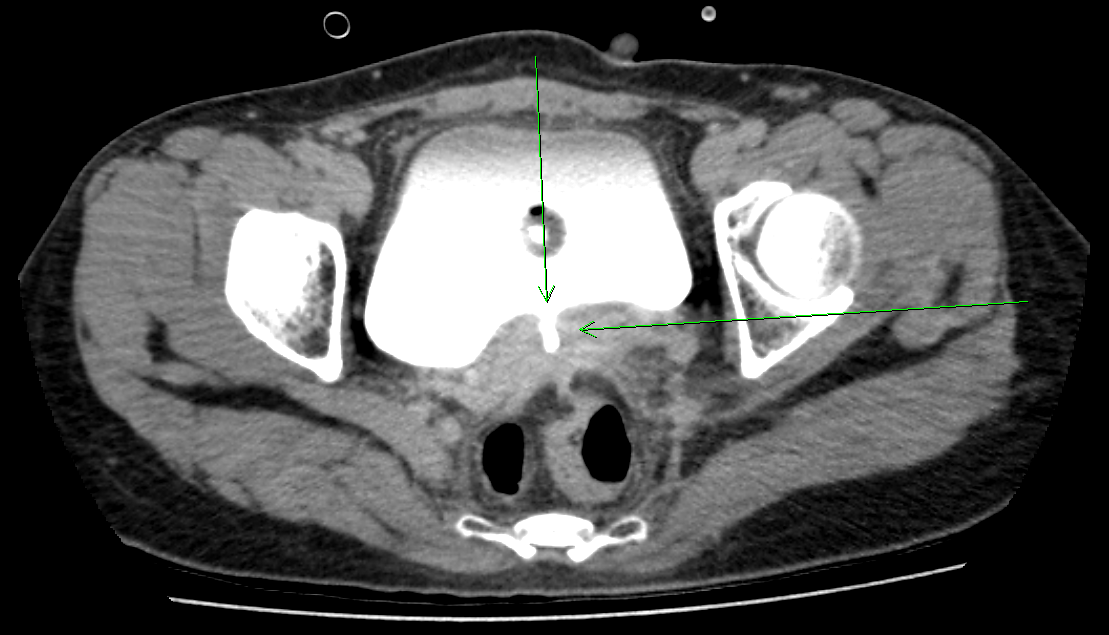  <Axial view of APCT^a^ showing vesico-vaginal fistula> |  |
|  |  | TLH^a^ | IIIB | **Sigmoidovaginal fistula was found on post-operative day 30. Laparoscopic lower anterior resection was done.**  Age: 42, height: 165 cm, weight: 57 kg (BMI^a^ 21.1)  Operative indication: adenomyosis, endometriosis  Uterus weight: 101 g, operation time: 1 hour 47 minutes   - Severe pelvic adhesion was seen during hysterectomy. Adhesiolysis was performed. - Patient was discharged on POD^a^ #3 uneventfully. - Patient visited the emergency department for abdominal pain and chilling sensation on POD^a^ #29 - APCT^a^ and colorectal x-ray study revealed sigmoidovaginal fistula. - Laparoscopic lower anterior resection (LAR) was performed. - Patient was discharged on POD^a^ #5 after LAR uneventfully.   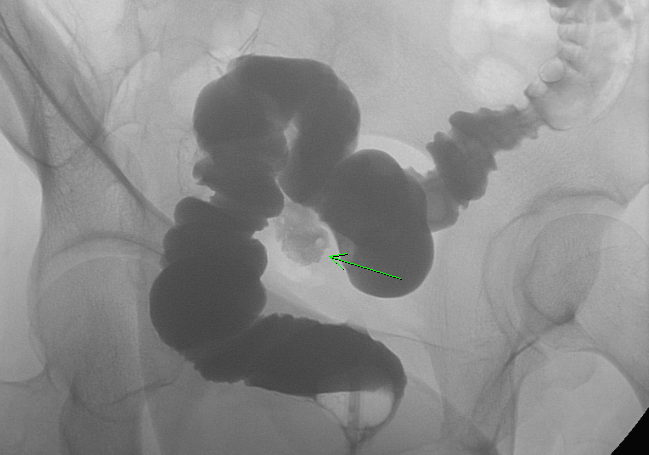  <Colorectal x-ray study revealed the leakage of contrast media> | |
| Postoperative bleeding | 5 | TLH^a^ | IIIB | **Diagnostic laparoscopy was performed and bleeding control was done.**  Age: 47, height: 163 cm, weight: 62 kg (BMI^a^ 23.7)  Operative indication: endometrial hyperplasia with atypia  Uterus weight: not measured, operation time: 2 hours 13 minutes   - Previous operation: cesarean section twice - Hysterectomy was done uneventfully. - About 6 hours after the operation, low blood pressure was checked (74/43 mmHg, baseline: 130/80 mmHg). Low hemoglobin was checked - 7.7 g/dL (pre-operative hemoglobin 12.4 g/dL) - Emergent diagnostic laparoscopy was done. Blood woozing from the stump area was seen. Coagulation and vessel ligation with suture was done. - Patient was discharged on POD^a^ #3 without further events. | |
|  |  | TLH^a^ | IIIB | **Diagnostic laparoscopy was performed and bleeding control was done.**  Age: 43, height 165 cm, weight: 58 kg (BMI^a^ 21.66)  Operative indication: leiomyoma  Uterus weight: not measured, operation time: 2 hours 16 minutes   - Hysterectomy was performed uneventfully. - About 15 hours after the operation, low blood pressure was checked (66/43 mmHg, baseline: 120/80 mmHg). Low hemoglobin was checked 7.6 g/dL (pre-operative hemoglobin 11.7 g/dL). - Emergent diagnostic laparoscopy was done. No focal site of bleeding was seen. Irrigation was performed. - Patient was discharged on POD #5 without further events. | |
|  |  | TLH^a^ | IIIB | **Diagnostic laparoscopy was performed and bleeding control was done.**  Age: 48, height: 162 cm, weight: 64 kg (BMI^a^ 24.7)  Operative indication: leiomyoma  Uterus weight: 760 g, operation time: 1 hour 20 minutes   - Obtaining clear visual field was difficult due to enlarged uterus (about 9 cm leiomyoma was located on the anterior wall of the uterus). - Low blood pressure (mean blood pressure 40 mmHg) was checked about 1 hour after the operation. Low hemoglobin was checked 6.6 g/dL (pre-operative hemoglobin 9.8 g/dL). - Emergent diagnostic laparoscopy was done. Blood woozing from the right uterine artery was seen. Coagulation with advanced energy device was done. - Patient was discharged on POD^a^ #4 without further events. | |
|  |  | TLH^a^ | IIIB | **Diagnostic laparoscopy was performed and bleeding control was done.**  Age: 61, height 150 cm, weight 38 kg (BMI^a^ 17.0)  Operative indication: leiomyoma  Uterus weight: not measured, operation time: 2 hours 2 minutes   - Hysterectomy was performed uneventfully. - About 6 hours after the operation, low blood pressure was checked (65/48 mmHg). Low hemoglobin was checked 6.8 g/dL (pre-operative hemoglobin 10.8 g/dL). - Emergent diagnostic laparoscopy was done. Blood woozing from the vaginal stump was seen. Coagulation and suture ligation was done. - Patient was discharged on POD^a^ #3 without further events. | |
|  |  | Cystectomy | IIIB | **Diagnostic laparoscopy was performed and bleeding control was done.**  Age: 30, height 167 cm, weight: 53 kg (BMI^a^ 19.2)  Operative indication: mature cystic teratoma  Operation time: 3 hours 7 minutes   - Bilateral ovarian cystectomy was done uneventfully (left 3 cm, right 8 cm mature cystic teratoma) - About 12 hours after the operation, low blood pressure (86/57 mmHg, baseline: 110/70 mmHg) was checked. Low hemoglobin was checked 7.2 g/dL (pre-operative hemoglobin 10.2 g/dL). - Emergent diagnostic laparoscopy was done. Blood woozing from the left ovary was seen. Electrocoagulation was performed. - Patient was discharged on POD^a^ #4 without further events. | |
| Vessel injury | 1 | TLH^a^ | IIIB | **Aorta injury during umbilical incision. Converted to laparotomy.**  Age: 49, height: 157 cm, weight: 47 kg (BMI^a^ 19.3)  Operative indication: adenomyosis  Uterus weight: not measured, operation time: 3 hours 52 minutes   - During the opening of the umbilicus by Hasson technique, aorta was injured by surgical knife when incising the fascial layer. Blood pressure dropped to 46/20 mmHg. Laparotomy was performed. Vascular surgeons identified bleeding focus on the abdominal aorta. Repair with pledget buttress polyprophylene was done. - Total abdominal hysterectomy was performed afterwards. Seven packs of RBC were transfused during the operation. - Patient was discharged on POD^a^ #5 without further events. | |
| Umbilical hernia | 3 | LAVH | IIIB | **Found 6 months after the operation. Did not require treatment.**  Age: 46, height: 164 cm, weight: 70 kg (BMI^a^ 25.7)  Operative indication: leiomyoma  Uterus weight: not measured, operation time: 2 hours 1 minute   - Hysterectomy was done uneventfully. - Umbilical port site was closed by continuous absorbable suture. - Patient claimed bulging sensation of the umbilicus 6 months after the operation. - Transabdominal ultrasonography revealed small defect of fascial layer (less than 1 cm). - Patient wanted conservative management. Serial follow-up of transabdominal ultrasonography for the next 2 years was done, which revealed no change in the size of the defect.   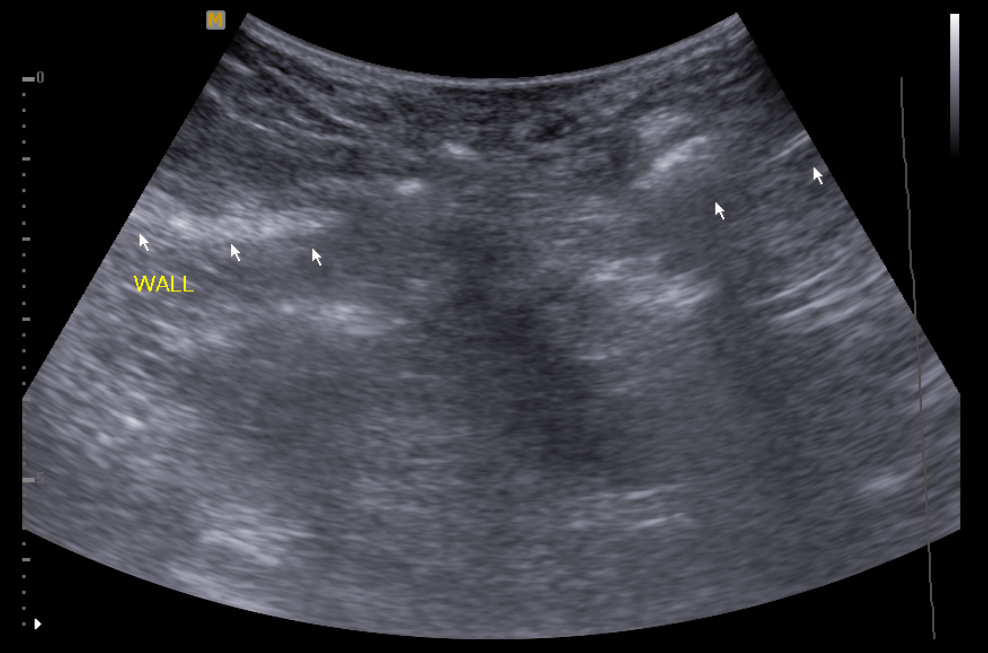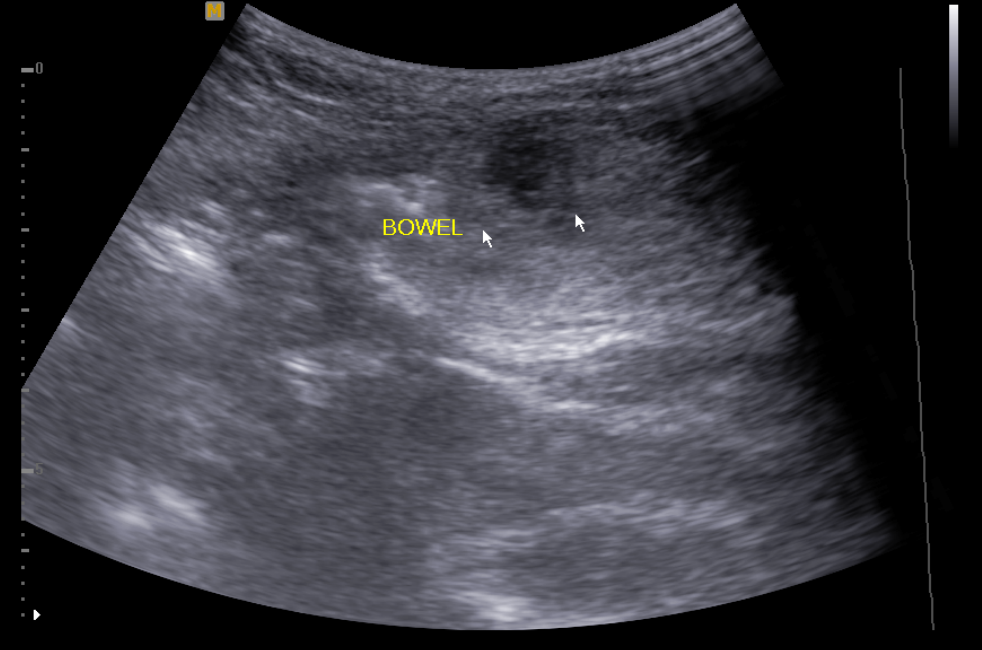  <Transabdominal ultrasonogrpahy at the umbilicus> | |
|  |  | TLH | IIIB | **Found 8 months after the operation. Repair was done without mesh placement.**  Age: 44, height: 159 cm, weight: 67 kg (BMI^a^ 26.6)  Operative indication: leiomyoma, adenomyosis  Uterus weight: 242 g, operation time: 1 hour 29 minutes   - Hysterectomy was done uneventfully. - Umbilical port site was closed by continuous absorbable suture. - Patient was discharged on POD^a^ #3 - Bulging of the umbilicus was noted 8 months after the operation. - APCT^a^ revealed incisional hernia. - About 6 cm defect of fascial layer at the umbilicus was noted. - Hernia repair with pants-over-vest tension suture was done. - Patient was discharged 3 days after the repair.   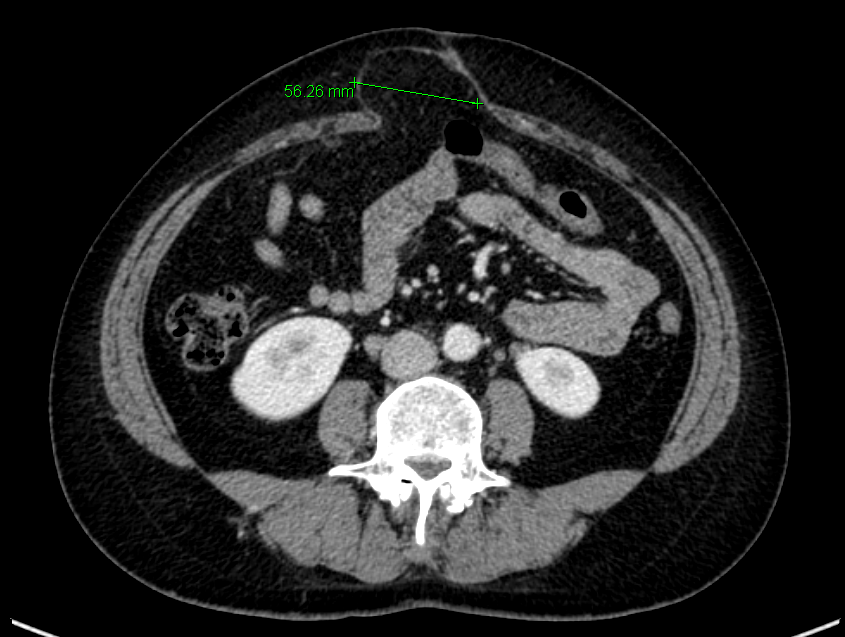  <Axial view of incisional hernia on APCT^a^> | |
|  |  | SH | IIIB | **Found 11 months after the operation. Repair was done without mesh placement.**  Age: 43, height: 145 cm, weight: 51 kg (BMI^a^ 24.3)  Operative indication: leiomyoma  Uterus weight: 370 g, operation time: 1 hour 20 minutes   - Subtotal hysterectomy was performed uneventfully. - Patient claimed bulging sensation at the umbilicus 11 months after the operation. - APCT^a^ revealed incisional hernia. - About 5 cm defect of fascial layer at the umbilicus was noted. - Hernia repair with pants-over-vest tension suture was done. - Patient was discharged 3 days after the repair.   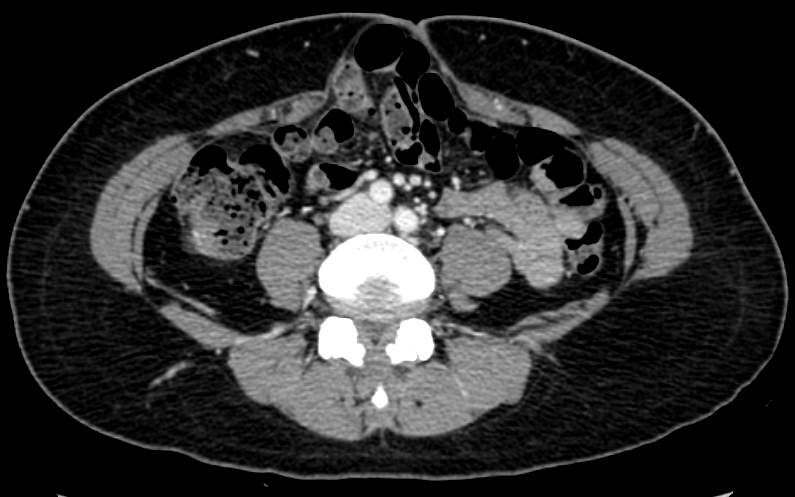  <Axial view of incisional hernia on APCT^a^> | |
| Vaginal vault evisceration | 1 | TLH | IIIB | **Occurred on postoperative day 68 after coitus. Primary repair was done.**  Age: 49, height 157 cm, weight: 45 kg (BMI^a^ 18.5)  Operative indication: leiomyoma  Uterus weight: 309 g, operation time: 1 hours 54 minutes   - Hysterectomy was done uneventfully. Patient was discharged on POD^a^ #3. - Patient visited the emergency department for lower abdominal pain after coitus. - Physical exam revealed 2 cm vaginal stump disruption and bowel prolapse. - Emergent repair of vaginal stump was done. Patient was discharged one day after the repair. | |
| Transfusion | 43 |  | II |  | |

^a^LAVH: laparoscopy-assisted vaginal hysterectomy, TLH: total laparoscopic hysterectomy, SH: subtotal hysterectomy, BMI: body mass index, POD: post-operative day, APCT: abdominopelvic computed tomography, WBC: white blood cell, CRP: c-reactive protein, ESR: erythrocyte sedimentation rate.
